# Risk factors of necrotizing enterocolitis in twin preterm infants

**DOI:** 10.1186/s12887-024-04701-6

**Published:** 2024-03-23

**Authors:** Ying-Ling Xie, Shu-Hua Lai, Su-Jia Liu, Wen-Long Xiu

**Affiliations:** Department of Neonatology, Fujian Maternity and Child Health Hospital College of Clinical Medicine for Obstetrics & Gynecology and Pediatrics, Fujian Medical University, Fuzhou, China

**Keywords:** Necrotizing enterocolitis, Twins, Preterm infants, Risk factors, Feeding intolerance

## Abstract

**Purpose:**

This study was aimed to investigate the risk factors of necrotizing enterocolitis (NEC) in twin preterm infants.

**Methods:**

The clinical data of 67 pairs of twin preterm infants admitted to the neonatal department of our hospital from January 2010 to December 2021 were retrospectively collected. One of the twins had NEC (Bell II and above) and the other twin without NEC. They were divided into NEC group and control group according to whether NEC occurred or not.

**Results:**

Univariate analysis showed that NEC was associated with congenital heart disease, small for gestational age, mild asphyxia at birth and feeding intolerance (*P* < 0.05).

**Conclusion:**

Occurrence of NEC was associated with congenital heart disease, small for gestational age, and asphyxia at birth. For twin preterm infants with congenital heart disease, small for gestational age, or asphyxia at birth, special attention should be paid to the occurrence of NEC to minimize and avoid the occurrence of NEC.

## Introduction

Necrotizing enterocolitis (NEC) is a common and serious disease with a high incidence and mortality in preterm infants [1–4]. About 90% of NEC cases occur in preterm infants and 10% in full-term infants [5, 6]. About 10–21% of preterm deaths are caused by NEC, and NEC remains a significant clinical problem [7, 8].

The incidence of NEC is significantly higher in twins compared to singleton pregnancies [2]. The fetus has similar genetics and intrauterine environment, and the identical twin fetus has exactly the same genetic material. To study the risk factors of NEC in twin preterm infants is more conducive to eliminating the influence of intrauterine and maternal pregnancy diseases. In this study, the risk factors of NEC in preterm twins were investigated on the basis of pairing twins analysis.

## Methods

This study retrospectively analyzed the clinical data of 67 pairs twin preterm infants treated in the neonatology department of our hospital from January 2010 to December 2021. One of the twins had NEC (Bell stage II and above) and the other twin without NEC. They were divided into NEC group and control group according to whether NEC occurred or not. The NEC diagnostic criteria and staging criteria are defined according to the improved Bell classification [9].

Inclusion criteria: (1) Preterm twin infants with gestational age less than 37 weeks; (2) Only one of the preterm twins had NEC, and the other of twins without NEC. Exclusion criteria: (1) Two of the twins with NEC; (2) Complicated with severe digestive tract malformations; (3) Complicated with genetic metabolic disease, (4) Death of one of the twins, 5.Incomplete clinical data.

### Data collection

1. Basic information: gestational age, birth weight, sex, birth order; 2. Perinatal risk factors: premature rupture of membranes, placenta abnormalities, and birth asphyxia; 3. Feeding status: microfeeding, feeding type, and breast milk fortifier; 4. Therapeutic measures: antibiotics, invasive procedures, vasoactive drugs, blood products, pulmonary surfactant and ibuprofen; 5. Complications: neonatal respiratory distress syndrome, neonatal sepsis, and congenital heart disease.

### Statistical analysis

SPSS 25.0 software was used for statistical analysis. Categorical variables were described as integers and percentages, and comparisons between groups were performed using Fisher’s exact test. Continuous variables were expressed as mean ± standard. Continuous variable with a normal distribution deviation were compared via the T-test. Continuous variable without a normal distribution deviation were compared via the Mann-Whitney U test. The risk factors of NEC were analyzed by univariate analysis and logistic regression analysis. A *P* < 0.05 was considered statistically significant.

## Results

In the NEC group, there were 42 males, 25 females with age of 31 (25, 36) weeks and birth weight of 1450 (700, 2160 ) g. In control group, there were 39 males, 28 females with age of 31 (25, 36) weeks and birth weight of 1480 (720, 2480 ) g. There was no significant difference in gestational age, birth weight, sex and birth order between the two groups (*P* > 0.05). (Table 1)

**Table 1:** Comparison of general data between the two groups

	NEC group	Control group	*P* value
Distribution of gestational age			
<28 weeks	10(14.9%)	10(14.9%)	1.000
28 ∼ < 32weeks	36(53.7%)	36(53.7%)	
32 ∼ 36weeks	21(31.3%)	21(31.3%)	
Distribution of weight			
<1000 g	8(11.9%)	10(14.9%)	0.744
1000 ∼ < 1500 g	28(41.8%)	24(35.8%)	
1500 ∼ < 2500 g	31(46.3%)	33(49.3%)	
≥ 2500 g	0	0	
Birth mass (g)	1450(1145,1765)	1480(1250,1810)	0.378
Male	42(62.7%)	39(58.2%)	0.596
Older of the twins	18(26.9%)	22(32.8%)	0.450

The incidence of congenital heart disease, small for gestational age, and mild asphyxia at birth in NEC group was higher than that in control group (*P* < 0.05). There were no significant differences in perinatal risk factors and complications such as premature rupture of membranes, placental abnormality, neonatal respiratory distress syndrome, and sepsis between the two groups (*P* > 0.05). There was no significant difference between the two groups in the main treatment measures such as pulmonary surfactant, vasoactive drugs, ibuprofen, caffeine, invasive ventilation, non-invasive ventilation, central venous catheterization, blood products and antibiotic therapy (*P* > 0.05). There was no significant difference between the two groups in the feeding conditions(*P* > 0.05). (Table 2)

**Table 2:** Comparison of risk factors between the two groups

	NEC group	Control group	*P* value
Premature rupture of membranes	16(23.9%)	17(25.4%)	0.841
Placental abnormality	1(1.5%)	1(1.5%)	-
Neonatal respiratory distress syndrome	21(31.4%)	20(29.9%)	0.851
Septicemia	5(7.5%)	6(9.0%)	0.753
Congenital heart disease			
Hemodynamically significant patent ductus arteriosus	5(7.5%)	1(7.5%)	0.033
Ventricular septal defect	3(4.5%)	0	
Small for gestational age	17(25.4%)	7(10.4%)	0.024
Birth Apgar score			
1 min	10 (8, 10)	10 (9, 10)	0.689
5 min	10 (10, 10)	10 (10, 10)	0.902
10 min	10 (10, 10)	10 (10, 10)	0.717
Mild asphyxia	17(25.4%)	7(10.4%)	0.024
Severe asphyxia	1(1.5%)	1(1.5%)	-
Using of pulmonary surfactant	22(32.8%)	20(29.9%)	0.710
Using of vasoactive drugs	32(47.8%)	27(40.3%)	0.384
Using of ibuprofen	11(16.4%)	15(22.4%)	0.382
Using of caffeine	22(32.8%)	17(25.4%)	0.342
Invasive ventilation	12(17.9%)	13(19.4%)	0.825
Noninvasive ventilation	48(71.6%)	41(61.2%)	0.456
Central vein catheterization	35(52.2%)	35(52.2%)	1.000
Blood transfusion	7(10.4%)	9(13.4%)	0.594
Gamma globulin infusion	34(50.7%)	35(52.2%)	0.863
Albumin infusion	33(49.3%)	34(50.7%)	0.863
Hemoglobin	124.03 ± 34.08	131.44 ± 38.00	0.243
Antibiotics	63(94.0%)	60(89.6%)	0.531
Situation of feeding			
Exclusive breastfeeding	1(1.5%)	3(4.5%)	0.919
Human milk fortifier	2(3.0%)	3(4.5%)	
Preterm milk feeding	30(44.8%)	28(41.8%)	
Deeply hydrolyzed milk powder feeding	14(20.9%)	12(17.9%)	
Mixed feeding	18(26.9%)	19(28.4%)	
Fasting	2(3.0%)	2(3.0%)	
Milk increase rate(ml/kg.d)	6.48 ± 4.74	6.59 ± 5.63	0.901
Minimal feeding	13(19.4%)	8(11.9%)	0.235

Univariate analysis showed that NEC was associated with congenital heart disease, small for gestational age, and mild asphyxia at birth(*P* < 0.05). (Table 3)

**Table 3:** Unifactor analysis of the cause of NEC

	B	Standard error	Wald	P	OR	95%CI
Congenital heart disease	2.192	1.076	4.151	0.042	8.949	1.087–73.690
Small for gestational age	1.070	0.488	4.800	0.028	2.914	1.119–7.587
Mild asphyxia at birth	1.070	0.488	4.800	0.028	2.914	1.119–7.587

## Discussion

NEC is a most common and severe gastrointestinal disease in preterm infants, with rapid progress, many complications, and high mortality [7]. How to reduce its morbidity and mortality is still a great challenge for neonatologists. So far, the cause of NEC has not been determined. Most researchers believe that the pathogenesis of NEC is multifactorial, usually associated with gene, preterm birth, low birth mass, lack of breastfeeding, ischemia, infection, and maternal diseases during pregnancy [10–12]. Due to the multi-factorial of NEC, there is a lot of uncertainty in identifying clear prevention strategies. The incidence of NEC in preterm twins was higher, and the intrauterine environment and genetic background of preterm twins were similar. In this study, the risk factors of NEC in preterm twins were investigated on the basis of pairing twins analysis.

Various studies have shown that infants with congenital heart disease have an increased risk of developing NEC [13, 14]. Congenital heart disease can cause diastolic dysfunction, diastolic blood flow reversal, heart failure, organ hypoperfusion, and target tissue hypoxia due to stolen blood, further increasing the risk of NEC. In this study, univariate analysis showed that the incidence of congenital heart disease in the NEC group was higher than that in the control group (11.9% vs.1.5%, *P* = 0.033). The incidence of infants who are small for gestational age increases. The study of Anne RP et al. [15] showed that compared with infants suitable for gestational age, the risk of NEC in neonate who were small for gestational age increases by more than two times.

Early neutropenia is more common in small gestational age infants than in normally growing neonate and is associated with an increased risk of NEC [16]. The immaturity of the digestive tract of preterm infants who are small for gestational age may be another factor leading to the development of NEC. In immature intestinal tissues, the tight connections between cells have not been fully formed, the mechanical barrier is immature, and toxic bacteria and various toxins are more likely to invade, leading to infection and NEC [17]. In this study, univariate analysis showed that the incidence of small gestational age was higher in the NEC group than in the control group (25.4% vs.10.4%, *P* = 0.024). Hypoxia plays an important role in the pathogenesis of NEC [18]. In this study, univariate analysis showed that the incidence of mild asphyxia at birth in the NEC group was higher than that in the control group (25.4% vs.10.4%, *P* = 0.024). It suggesting that asphyxia at birth was a risk factor for the development of NEC.

Preterm infants are prone to be feeding intolerance and NEC due to the immature anatomical and functional development of the gastrointestinal tract, insufficient secretion of gastrointestinal hormones, poor swallowing coordination, intestinal flora imbalance, and the disease state. Feeding intolerance is a common symptom of NEC, which often leads to the interruption of enteral feeding, the delay of full enteral feeding, and the prolongation of hospital stay [19]. Although there was no significant difference in feeding state between the NEC group and the Control group in this study, in the NEC group, the patients fed with preterm milk had the highest proportion, followed by mixed feeding and deeply hydrolyzed milk powder feeding, and the patients fed with breast milk had the lowest proportion. The reason may be that the osmotic pressure of preterm milk is higher than that of breast milk and deeply hydrolyzed milk powder feeding. Preterm milk is more difficult to absorb and more likely to cause NEC. Therefore, we should pay special attention to observe the feeding of preterm infants. Breast milk is very important at this stage.

Previous studies have showed that premature rupture of membranes, placental abnormalities, neonatal respiratory distress syndrome, sepsis and severe asphyxia were mostly associated with NEC [20–22]. Our study also showed that asphyxia was a risk factor for NEC, which was consistent with their study. However, the results of our study show that NEC was not associated with premature rupture of membranes, placental abnormalities, neonatal respiratory distress syndrome, and sepsis. This may be due to the single-center nature of our study with small sample size, and a larger sample size may be needed to show differences in these factors. Therefore, multi-center studies with large samples are the next step to be considered.

The study had some limitations. (1) This study was a single-center study. (2) The sample size of this study was small. (3) This was a retrospective study.

## Conclusion

Occurrence of NEC of twin preterm infants was associated with congenital heart disease, small for gestational age, and asphyxia at birth. For twin preterm infants with congenital heart disease, small for gestational age, or asphyxia at birth, special attention should be paid to the occurrence of NEC to minimize and avoid the occurrence of NEC.

## Data Availability

The original contributions presented in the study are included in the article, further inquiries can be directed to the corresponding author.

## Abbreviations

NEC: Necrotizing enterocolitis
